# Rib stress fracture in a male adaptive rower from the arms and shoulders sport class: case report

**DOI:** 10.3325/cmj.2011.52.644

**Published:** 2011-10

**Authors:** Tomislav Smoljanović, Ivan Bojanić, Courtney L. Pollock, Radovan Radonić

**Affiliations:** 1 Department of Orthopaedic Surgery, University Hospital Center Zagreb, School of Medicine, University of Zagreb, Zagreb, Croatia; 2 Graduate Program in Rehabilitation Science, Faculty of Medicine, University of British Columbia, Vancouver, British Columbia, Canada; 3 Department of Internal Medicine, University Hospital Center Zagreb, School of Medicine, University of Zagreb, Zagreb, Croatia

## Abstract

Adaptive rowing is rowing or sculling for rowers with a physical disability. It debuted at the Paralympic Games in 2008. In order to ensure an equitable playing field, rowers with similar levels of physical function and disability are classified into different sport classes for competition. Rowers with an inability to use a sliding seat and impaired trunk function resulting in an inability to perform trunk forward and backward lean via hip flexion/extension are assigned to the Arms and Shoulders (AS) class. AS rowers have to use a chest strap set immediately below the chest in order to localize any trunk movement in AS class. Conditions created by adaptations of rowing equipment and technique within the AS class create unique stresses on the upper thoracic region. The following case report demonstrates how etiology and management of a rib stress fracture in an AS rower differs in comparison to able-body rowers. Of significant importance were the limitations imposed on the rower’s ability to maintain rowing-specific fitness, due to the nature of the rib stress fracture and requirement to decrease force transmission through the ribs for several weeks. The rower’s gradual return to full training was further impacted by obligatory use of the chest strap, which directly applied pressure over the injured area. Protective orthosis for the chest was designed and applied in order to dissipate pressure of the chest strap over the thorax during rowing (most importantly at the catch position) both on the ergometer and in the boat.

Adaptive rowing at the Paralympic level is rowing or sculling for rowers with a physical disability (Table 1) (1-3). A Medline search using key words for adaptive or Paralympic rowing revealed an absence of publications exploring the injuries associated with adaptive rowing specific to the adaptation of the rowing technique to the functional abilities of the rower.

**Table 1:** International competitive adaptive rowing sport class and boat class description (1,2)

Adapted Rowing Sport Class Boat Class	Description
Arms and Shoulders women or men Rowers compete in single sculls (1x)	Rowers with inability to use a sliding seat and limited trunk function resulting in an inability to perform trunk forward and backward lean via hip flexion and extension (bodyswing)
Trunk and Arms Rowers compete in double sculls (2x)	Rowers who have functional use of trunk movement to create bodyswing, however, are unable to use the sliding seat to propel the boat because of significantly weakened function or mobility of the lower limbs
Leg Trunk and Arms Rowers compete in coxed fours boats (4+) referring to 4 rowers and one coxswain to steer the boat	Rowers with a verifiable and permanent disability (meeting a set minimal disability) who have rowing-specific functional use of their legs, trunk and arms, utilizing the sliding seat to propel the boat

## Case report

A 23-year-old Arms and Shoulders class (AS) rower presented with abrupt onset, two-day history of left anterolateral chest pain on August 19, 2010.

The already present rower’s permanent disability was T9 complete paraplegia as a result of trauma occurring in 2005. He started rowing in February 2010, 3 times per week without previous experience in any sport for athletes with or without physical disabilities. Initial training sessions were performed on a rowing ergometer with a fixed seat and chest strap made of non-elastic material. After the basic techniques of rowing were acquired, training sessions were continued in an adaptive single scull boat beginning in April. From April onwards, the athlete performed two rowing-specific training sessions either on water or on an ergometer and two strength-training sessions in the gym. Beginning in June, training shifted to rowing-specific training five sessions per week (comprised of approximately 8 km per session to a maximum of 12 km per session). He joined the National Team in July and began training for the World Rowing Championships in November 2010. Training sessions consisted of shorter distances, but greater intensity (2 × 1000 m, 3 × 500 m, or sprint distances in pyramid design 250 m, 500 m, 750 m, 500 m, 250 m), which were introduced between sessions of steady state rowing focusing on rowing technique. After five weeks of increased frequency, volume, and intensity of training, the rower noted left-side chest dull pain while rowing and during deep inspiration.

On palpation, there was marked tenderness over the ninth rib in the midclavicular line with discomfort in the whole region of the left hemithorax. Radiographs demonstrated no signs of fracture at the time. Bone scan showed a well defined focus of increased isotope uptake in the anterolateral part of left ninth rib, confirming a stress injury in this area.

**Figure 1:**
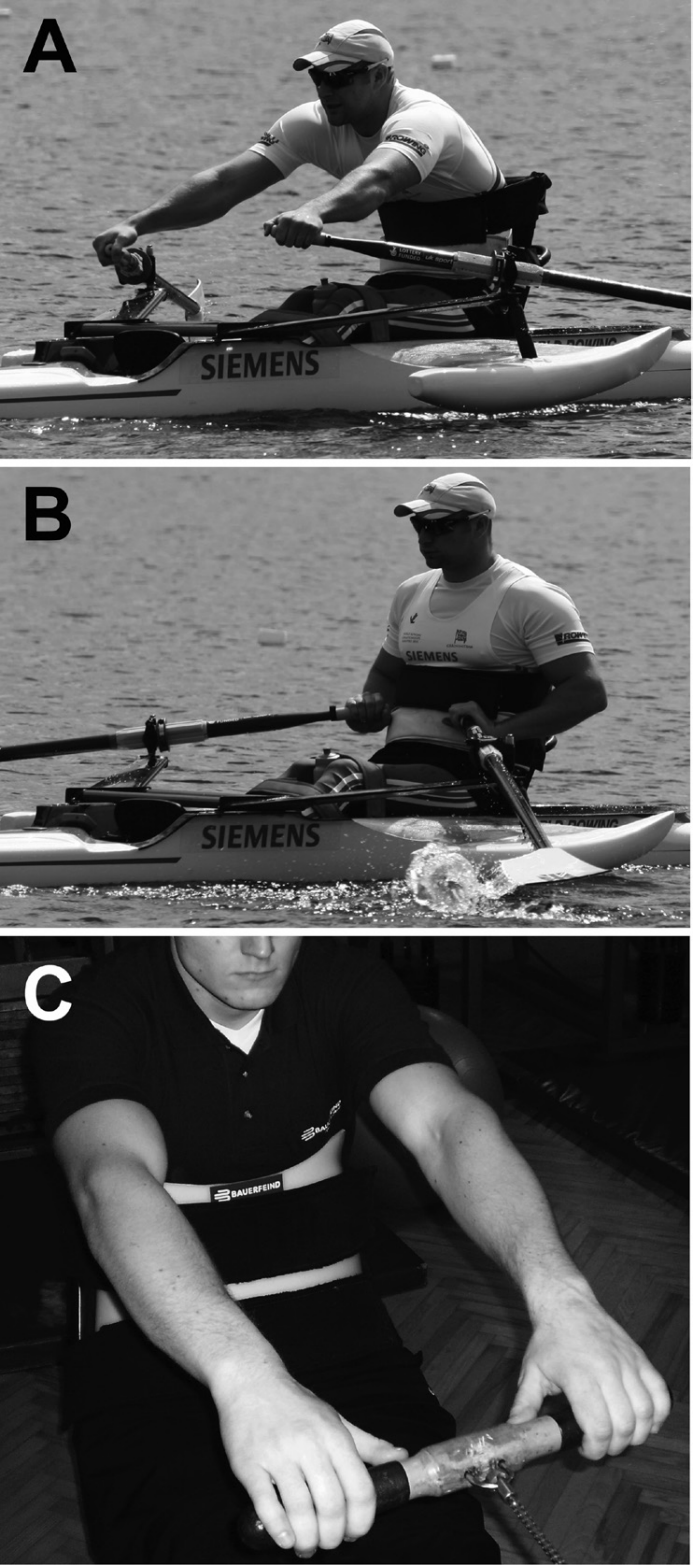
A rower in the Arms and Shoulders class is able to apply force predominantly using the arms and/or shoulders. The rowers use a single sculling boat with a fixed seat and stabilizing pontoons attached to the riggers. The rowers use a strap that must be secured to the seat back, and around the torso just below the nipple line or the breasts. The strap is tight enough to restrict trunk movement while not restricting breathing. It must be a minimum width of 50 mm and made of non-elastic material. (**A**) Catch-point at which the blade enters the water. The arms are outstretched, shoulders are forward flexed, and trunk is leaned against the chest strap. (**B**) Finish-point at which the blades exit the water. The arms are drawn inwards and trunk is leaned against the back rest. (**C**) A chest-protective orthosis (Bauerfeind d.o.o., Zagreb, Croatia) was made of thermoformed plastic (polypropylene) and lined with energy-absorbing foam (plastazote) in order to decrease pressure on the rib cage by increasing the strap stress distribution area across a wider area during rowing on ergometer or in the boat.

Management of the stress fracture consisted of 5 weeks cessation of rowing until there was no tenderness on palpation and deep inspiration. During that time, physical fitness was not maintained to avoid aggravating the injury. The rower’s gradual return to full training was slow, partly because of decreased fitness, and partly because of obligatory use of the chest strap, which directly applied pressure over the injured area. A protective orthosis (Bauerfeind d.o.o., Zagreb, Croatia) was designed and applied in order to decrease pressure from the chest strap over the thorax during rowing (particularly at the catch position) both on the ergometer and in the boat (Figure 1C). Orthoses were successfully used by several British adaptive rowers suffering from rib stress fractures (personal communication with Mr. Simon Goodey). However, the adaptive rower presented here was not able to prepare well for the shortly upcoming World Rowing Championships and did not participate.

## Discussion

Adaptive rowers from AS class may be more susceptible to rib stress fractures and other overuse injuries as the nature of their physical disability and associated adjustment in rowing technique increases stress on the remaining functional parts of their musculoskeletal system.

Rowers without physical disability reporting occurrence of rib stress fractures were between 19 and 33 years old and 86% of them performed at the elite level (4). In most cases, there was a history of an increase in training intensity/duration associated with overuse mechanism of injury or a change of rowing technique leading up to the injury (5). In adaptive rowing, different areas of the body experience higher force transmission depending on the technique utilized in comparison to able-body rowing. In the Leg, Trunk, and Arms class (LTA), as in able-bodied rowing, the extension of the lower extremities is the main producing force. The forces generated by the legs are further increased and transferred to the oar by extension of the trunk and flexion of the upper extremities (6). In the Trunk and Arms class, the spine and pelvis, as a somewhat rigid lever together with the upper extremities, generate and transfer forces during the stroke as rowers utilize body swing and arm pull as their rowing stroke technique. In the AS class, only the upper extremities, together with the associated upper thorax, generate and transfer forces to the oar. Potential for overuse injury related to technique is further influenced by use of increased stroke rate, weight of the boat, and length of time of the race. Able-bodied or LTA rowers usually do fewer than 40 strokes per minute during the majority of the race, while some AS rowers can perform at >45 strokes per minute depending on their boat rigging. Finally, the goal of maximal stroke length in the AS class demands maximal reach, commonly resulting in loading during the initial to mid-drive phase through large degrees of shoulder flexion angle excursion. Increased shoulder flexion angle excursion during the initial drive phase in able-bodied rowers has been associated with rib stress fractures (7).

Modified pattern of injuries in adaptive rowing may also be related to adaptation of equipment. Development of only three sport classes for rowers with a wide range of physical disabilities results in adaptive rowing being a very competitive sport. On the other hand, a wide range of disabilities within each of the three classes may give some of the athletes an advantage over others. Equipment regulations were made in order to minimize the differences within the classes. For example, chest strap level (originally introduced for safety reasons of stabilizing the trunk of AS rowers) was regulated immediately below the breasts to limit trunk movement in AS rowers. AS class rowers are able to flex over the strap as much as possible in order to increase the length of their stroke. Although the increase in length of the stroke is just a few centimeters, the gain can be quite significant over the course of the race. However, the “flexing” over the strap increases pressure on ribs in that area, creating a pivot point. The pressure occurring in the area of the strap during race rate rowing is further influenced by the momentum utilized during the recovery of the stroke to reach their catch position quickly. For these reasons, an orthosis was introduced to distribute loading at the catch position across a wide area of padding.

Finally, to reach an A final, and even more to win a medal, a rower must take the same approach to adaptive rowing as to any other high-performance sport. In this way, one can easily fall into the trap of a well-known training mistake “too soon, too much” (8), as was the case with this rower. Overuse injury management requires periods of time with altered training to maintain optimal conditioning. Unfortunately, training methods to maintain conditioning are limited by the functional limitations of adaptive rowers (9). This significantly impacted this rower’s ability to return to rowing at a performance level consistent with international competition. Future research is required to address training associated with adaptive rowing with regards to prevention of overuse injury, such as rib stress fractures, and optimal management of these injuries when they occur.

## References

[1] FISA. Adaptive rowing regulations. 2010. Available from: http://www.worldrowing.com/medias/docs/media_360593.pdf Accessed: December 11, 2010.

[2] FISA. Classification guidelines for adaptive rowers. 2010. Available from: http://www.worldrowing.com/medias/docs/media_360544.pdf Accessed: December 11, 2010.

[3] Smoljanovic T, Bojanic I, Morrison J, Chung OM, Jelic M, Pecina M (2008). Adaptive rowing – rowing or sculling for rowers with a disability. Hrvatski Športskomedicinski Vjesnik..

[4] Smoljanovic T, Bojanic I (2007). Ewing sarcoma of the rib in a rower: a case report.. Clin J Sport Med.

[5] Warden SJ, Gutschlag FR, Wajswelner H, Crossley KM (2002). Aetiology of rib stress fractures in rowers.. Sports Med.

[6] Pollock CL Jones IC Jenkyn TR Ivanova TD Garland SJ Changes in kinematics and trunk electromyography during a 2000 m race simulation in elite female rowers Scand J Med Sci Sports 2010Dec 3. [Epub ahead of print] 10.1111/j.1600-0838.2010.01249.x 21129036

[7] Vinther A, Kanstrup IL, Christiansen E, Alkjaer T, Larsson B, Magnusson SP (2006). Exercise-induced rib stress fractures: potential risk factors related to thoracic muscle co-contraction and movement pattern.. Scand J Med Sci Sports.

[8] Pecina M, Bojanic I, editors. Overuse injuries of the musculoskeletal system. 2nd ed. Boca Raton: CRC Press; 2003.

[9] Jacobs PL, Nash MS (2004). Exercise recommendations for individuals with spinal cord injury.. Sports Med.

